# A Novel Ferroptosis-Related Gene Signature Predicts Recurrence in Patients With Pancreatic Ductal Adenocarcinoma

**DOI:** 10.3389/fmolb.2021.650264

**Published:** 2021-09-23

**Authors:** Zengyu Feng, Peng Chen, Kexian Li, Jianyao Lou, Yulian Wu, Tao Li, Chenghong Peng

**Affiliations:** ^1^ Department of General Surgery, Ruijin Hospital, Shanghai Jiaotong University School of Medicine, Shanghai, China; ^2^ Research Institute of Pancreatic Diseases, Shanghai Jiaotong University School of Medicine, Shanghai, China; ^3^ Department of General Surgery, The Second Affiliated Hospital, School of Medicine, Zhejiang University, Hangzhou, China

**Keywords:** pancreatic ductal adenocarcinoma, ferroptosis, prognostic model, bioinformatics, recurrence, prognosis

## Abstract

**Background:** Recurrence after surgery is largely responsible for the extremely poor outcomes for patients with pancreatic ductal adenocarcinoma (PDAC). Ferroptosis is implicated in chemotherapy sensitivity and tumor recurrence, we aimed to find out survival-associated ferroptosis-related genes and use them to build a practical risk model with the purpose to predict PDAC recurrence.

**Methods:** Univariate Cox regression analysis was conducted to obtain prognostic ferroptosis-related genes in The Cancer Genome Atlas (TCGA, N = 140) cohort. Multivariate Cox regression analysis was employed to construct a reliable and credible gene signature. The prognostic performance was verified in a MTAB-6134 (N = 286) validation cohort and a PACA-CA (N = 181) validation cohort. The stability of the signature was tested in TCGA and MTAB-6134 cohorts by ROC analyses. Pathway enrichment analysis was adopted to preliminary illuminate the biological relevance of the gene signature.

**Results:** Univariate and multivariate Cox regression analyses identified a 5-gene signature that contained CAV1, DDIT4, SLC40A1, SRXN1 and TFAP2C. The signature could efficaciously stratify PDAC patients with different recurrence-free survival (RFS), both in the training and validation cohorts. Results of subgroup receiver operating characteristic curve (ROC) analyses confirmed the stability and the independence of this signature. Our signature outperformed clinical indicators and previous reported models in predicting RFS. Moreover, the signature was found to be closely associated with several cancer-related and drug response pathways.

**Conclusion:** This study developed a precise and concise prognostic model with the clinical implication in predicting PDAC recurrence. These findings may facilitate individual management of postoperative recurrence in patients with PDAC.

## Introduction

Pancreatic ductal adenocarcinoma (PDAC) is one of the most aggressive gastrointestinal tumors with a 5-years survival rate not exceeding 9% (Siegel et al., 2019). Surgery combined with adjuvant chemotherapy is the standard treatment for resectable PDAC (Garrido-Laguna and Hidalgo, 2015), and it significantly elevates the 5-years survival rate to 20–25% (Hackert et al., 2016). Unfortunately, majority of PDAC patients miss the chance for this regimen owing to atypical and unspecific symptoms at an early, resectable stage (Heinemann and Boeck, 2008; Sharma et al., 2011; Rombouts et al., 2015). Furthermore, even after radical resection, most patients will develop recurrence (Vincent et al., 2011), and patients with tumor recurrence have significantly decreased overall survival rates compared with patients without tumor recurrence (Breidert et al., 2012). Under this grim circumstance, novel development of recurrence risk prediction models is urgently needed for clinicians to manage patients by more tailored therapeutic strategies and postoperative surveillance.

Ferroptosis is an iron-dependent form of non-apoptotic cell death, which is featured with lipid peroxidation (Dixon et al., 2012a). Triggering ferroptosis has shown potential therapeutic value in oncology (Shan et al., 2020), especially for eradicating aggressive malignancies that are refractory to conventional treatments (Roh et al., 2017; Shin et al., 2018; Gao et al., 2019). In PDAC, activation of ferroptosis by knockdown ARF6 gene could enhance the sensitivity to gemcitabine (Ye et al., 2020), the cornerstone drug of chemotherapy for PDAC (Heinemann, 2001). Anticancer effects of ferroptosis inducers on PDAC have also been reported (Eling et al., 2015). In addition, Tang et al. found that diminished ferroptosis sensitivity is associated with decreased immune activity and robust gemcitabine resistance in PDAC (Tang et al., 2020). These previous studies revealed that ferroptosis represents an under-explored source of prognostic factors that could be used to predict tumor recurrence.

In this study, we analyzed the correlation between ferroptosis-related genes with recurrence-free survival (RFS) of patients, and developed a 5-gene risk prediction model with satisfactory predictive performance in both training and validation datasets. The proposed signature outperformed clinical parameters and previous reported models in predicting tumor recurrence. In addition, this signature is positively correlated with cell division and DNA replication. The 5-gene signature is promising to help to make individual treatments and has potential to be implemented for clinical practice.

## Materials and Methods

### Data Collection

The three PDAC cohorts included in this study for survival analyses were the MTAB-6134 cohort (N = 286), PACA-CA cohort (N = 181) and TCGA cohort (N = 140). TCGA cohort was used as the training set, while MTAB-6134 cohort and PACA-CA cohort were adopted for external validation. For samples in TCGA cohort, gene expression data and corresponding clinical data were downloaded from the TCGA hub at UCSC Xena (https://tcga.xenahubs.net). Microarray data and clinical information of MTAB-6134 cohort was obtained from ArrayExpress (https://www.ebi.ac.uk/arrayexpress/) database. In the cases of PACA-CA cohort, normalized RNA-sequencing data and survival data were downloaded from the International Cancer Genome Consortium (ICGC, https://icgc.org/) database. For TCGA cohort expression profiles, the gene expression profile was measured experimentally using the Illumina HiSeq 2000 RNA Sequencing platform by the University of North Carolina TCGA genome characterization center, and the gene-level transcription was estimated by log2(x+1) transformed RSEM normalized count. For MTAB-6134, Affymetrix Human Genome U219 Array were performed to annotate transcription profiles, and the expression levels can be fetched, too. For PACA-CA, ICGC portal provided us with convenient access to fetch normalized expression profiles. Patients whose clinical information was incomplete or whose histopathological type was not PDAC were strictly removed. Clinical RFS data was available in all of these three cohorts, and patients with a RFS < 1 month were excluded. In addition, we also downloaded gene expression data of GSE15471 cohort and GSE16515 cohort from the Gene Expression Omnibus (GEO, https://www.ncbi.nlm.nih.gov/ geo/) database, aiming to assess the distribution of risk score in PDAC tissues and matched adjacent normal tissues.

### Construction of the Ferroptosis-Related Gene Signature for Prognostic Prediction

260 ferroptosis-related gene were retrieved and obtained from the FerrDb database (http://www.zhounan.org/ferrdb) (Zhou and Bao, 2020). In the training TCGA cohort, ferroptosis-related gene that were significantly associated with the RFS of PDAC patients were screened through using the univariate Cox regression analysis. The following multivariate Cox regression analysis was employed to select an optimal prognostic model with the minimum Akaike Information Criterion value. Based on the expression level and corresponding coefficient of each prognostic gene derived from the multivariate Cox regression analysis, the risk score of every patient in all of three cohorts was computed.

### Prognostic Performance of the Ferroptosis-Related Gene Signature

According to the best cut-off value determined by the X-tile software (Camp et al., 2004), patients in both the training and validation cohorts were assigned to a low- or a high-risk group. The differences in OS time between the two groups were estimated by the Kaplan–Meier survival curves. To determine whether our signature provided improved survival prediction, ROC analyses were performed on the ferroptosis-related gene signature, clinical indicators and two previously reported gene signatures predicting PDAC recurrence (Shi et al., 2017; Kim et al., 2019a).

### Functional Enrichment Analyses

Genes correlated with the risk score were identified using Spearman correlation analysis in MTAB-6134 cohort (*p* < 0.05) and TCGA cohort (*p* < 0.05). The top 1,000 positively correlated genes were selected and then submitted to the Metascape database (Zhou et al., 2019) for function annotation and pathway enrichment.

### Statistical Analysis

The statistical analysis and graphical work were finished in the R environment (version 3.5.2). Cox regression analyses and K-M survival curves were conducted by the “survival” package. The ROC curves for prognosis were plotted by the “survivalROC” package. Boxplots were generated from the “ggpubr” package. Calibration curves were derived from the “rms” package. A two-sided log-rank *p* < 0.05 was considered significant.

## Results

### Construction of the Prognostic Ferroptosis-Related Signature for PDAC

**FIGURE 1:**
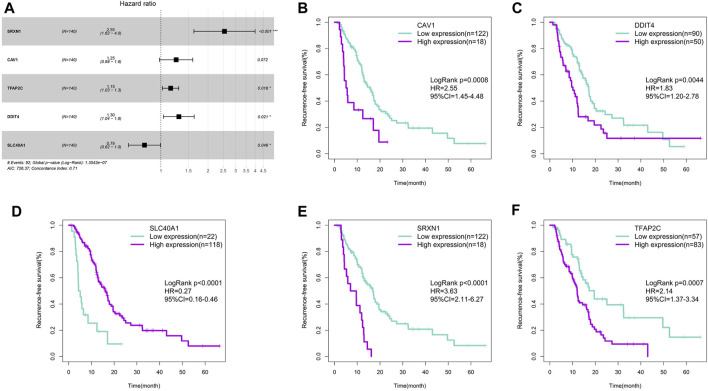
Construction of the ferroptosis-related gene signature. **(A)** Forest plot of the five ferroptosis-related genes. **(B)** Survival cure for CAV1. **(C)** Survival cure for DDIT4. **(D)** Survival cure for SLC40A1. **(E)** Survival cure for SRXN1. **(F)** Survival cure for TFAP2C.

The univariate Cox regression analysis screened 21 of 260 ferroptosis-related genes that were significantly associated with RFS in the TCGA training cohort (*p* < 0.05). Subsequently, the multivariate Cox regression analysis was implemented to generate a prognostic model. Finally, five ferroptosis-related genes with high correlation with RFS were selected to make up the signature, and the HRs, 95%CIs, and *p* values of these five genes were illustrated in Figure 1A. Each of the five genes could effectively stratify PDAC patients with different RFS time in the TCGA training cohort (*p* < 0.05, Figures 1B–F). Among the five genes, CAV1, DDIT4, SRXN1 and TFAP2C were indicative of better survival while SLC40A1 was associated with worse survival. According to the expression of these five genes, we developed a risk-score formula: Risk score = (0.224696×expression value of CAV1)+(0.265016×expression value of DDIT4)-(0.24004×expression value of SLC40A1)+(0.934599×expression value of SRXN1)+(0.144059×expression value of TFAP2C).

### Distribution of the Risk Score

**FIGURE 2:**
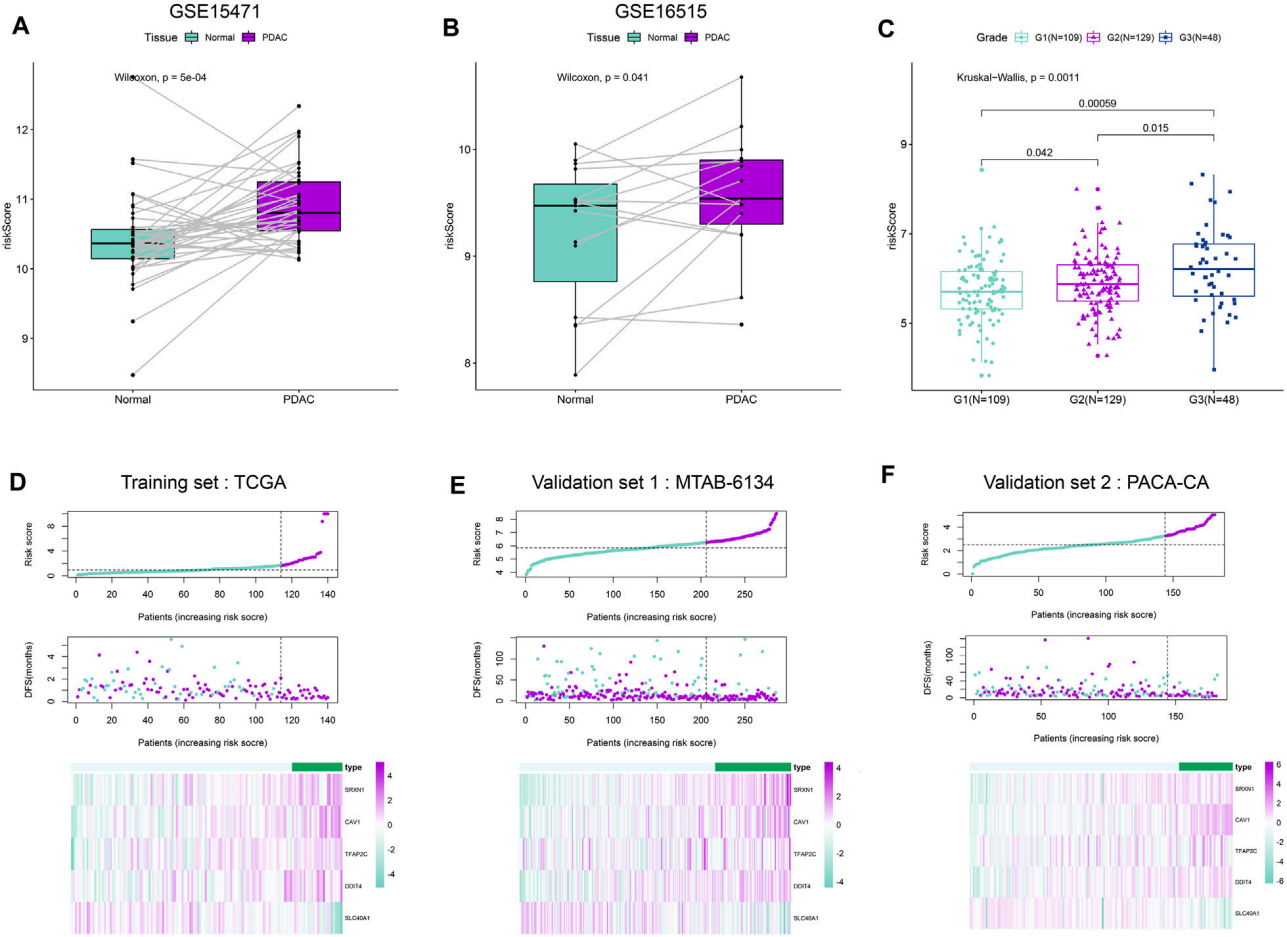
Distribution of the risk score. **(A**,**B)** Distribution of risk scores in PDAC tissues and paired adjacent normal tissues in GSE15471 dataset and GSE16515 dataset. **(C)** Distribution of risk scores with respect to histological grade. We have calculated the *p* values correction using “Benjamini & Hochberg” method, and the adjusted *p* values are 0.00177(G1 vs. G3), 0.042(G1 vs. G2) and 0.0225(G2 vs. G3), respectively. **(D**–**F)** From top to bottom are the risk score distribution, survival status distribution, and heat map analysis of five genes in three independent cohorts.

We first calculated the risk score of samples in GSE15471 and GSE16515 cohorts. These two cohorts contained PDAC and paired adjacent normal samples. As shown in Figures 2A,B, PDAC tissues exhibited a remarkably elevated risk score compared with adjacent normal tissues, suggesting the hazardous role of risk score. Meanwhile, we observed that the risk score increased with the increase of histological grade in MTAB-6134 cohort (Figure 2C). This finding indicated that risk score is associated with tumor malignancy. According to the optimal cut-off value of risk score computed by X-tile software, patients were divided into high- and low-risk groups. The distribution of the risk scores, survival status, and heatmap for five genes in both training and validation samples were illustrated in Figures 2D–F. Low-risk-score patients had a dramatically decreased mortality rate compared with high-risk-score patients in these three cohorts.

### Prognostic Validation of the Ferroptosis-Related Signature

**FIGURE 3:**
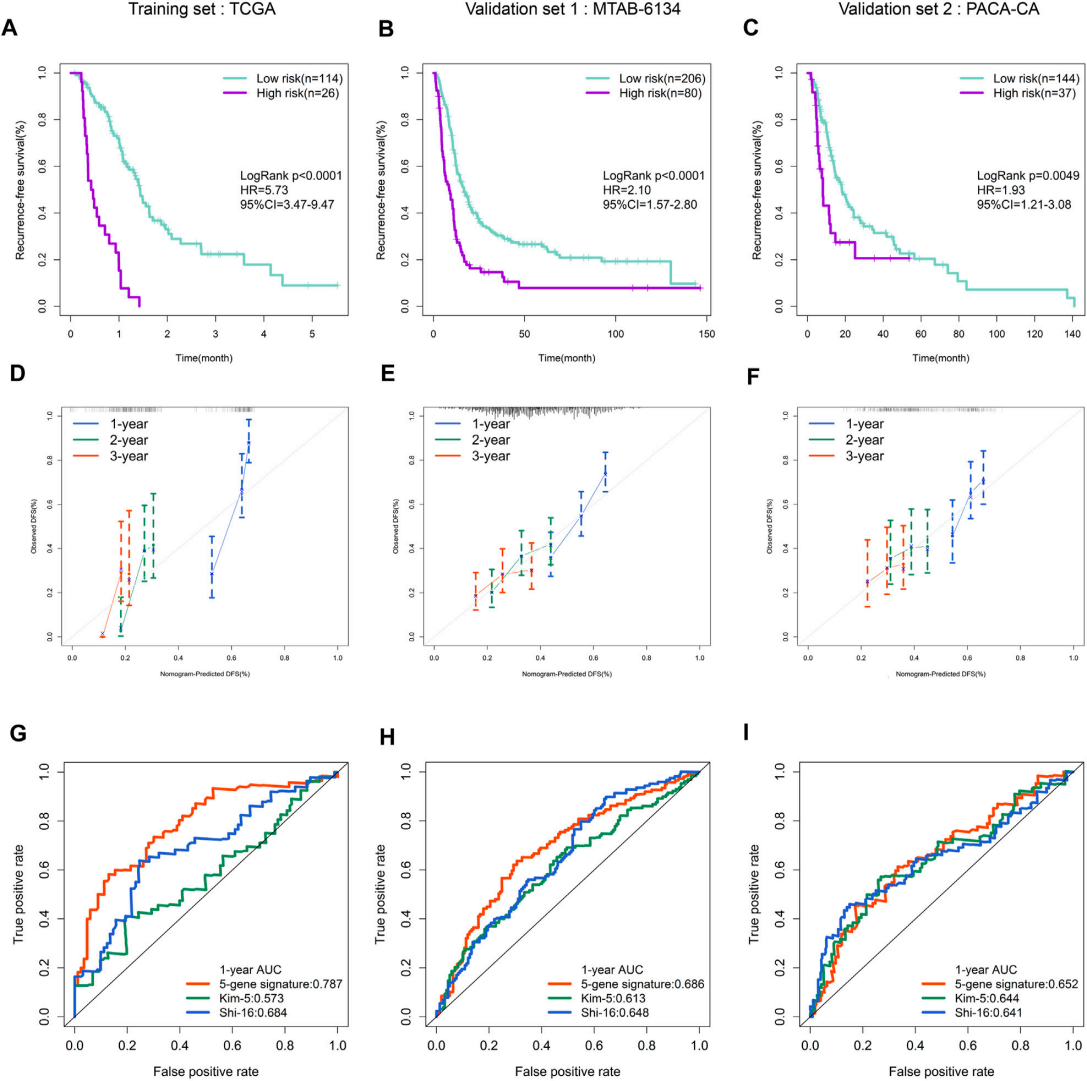
Prognostic validation of the ferroptosis-related gene signature. **(A**–**C)** K–M curves evaluating the RFS between low- and high-risk groups in three independent cohorts. **(D**–**F)** Calibration curves for risk score in three independent cohorts. **(G**–**I)** ROC curves of risk models in three independent cohorts.

The Kaplan–Meier curve demonstrated that patients in high-risk group had a significantly shorter RFS than patients in low-risk group in both training and validation cohorts (Figures 3A–C). The calibration curves showed that the RFS predicted by the ferroptosis-related signature were in good accordance with the observed RFS (Figures 3D–F). ROC curves revealed that this signature had moderate accuracy for predicting 1-year RFS, as the area under the curve (AUC) value was 0.787, 0.686 and 0.652 in three cohorts, respectively (Figures 3G–I). In addition, the AUC value of our signature was higher than that of two previous reported gene signatures with recurrence predictive ability. This finding indicated that the proposed ferroptosis-related signature could provide improved outcome prediction.

### Subgroup Analyses of the Ferroptosis-Related Signature

**FIGURE 4:**
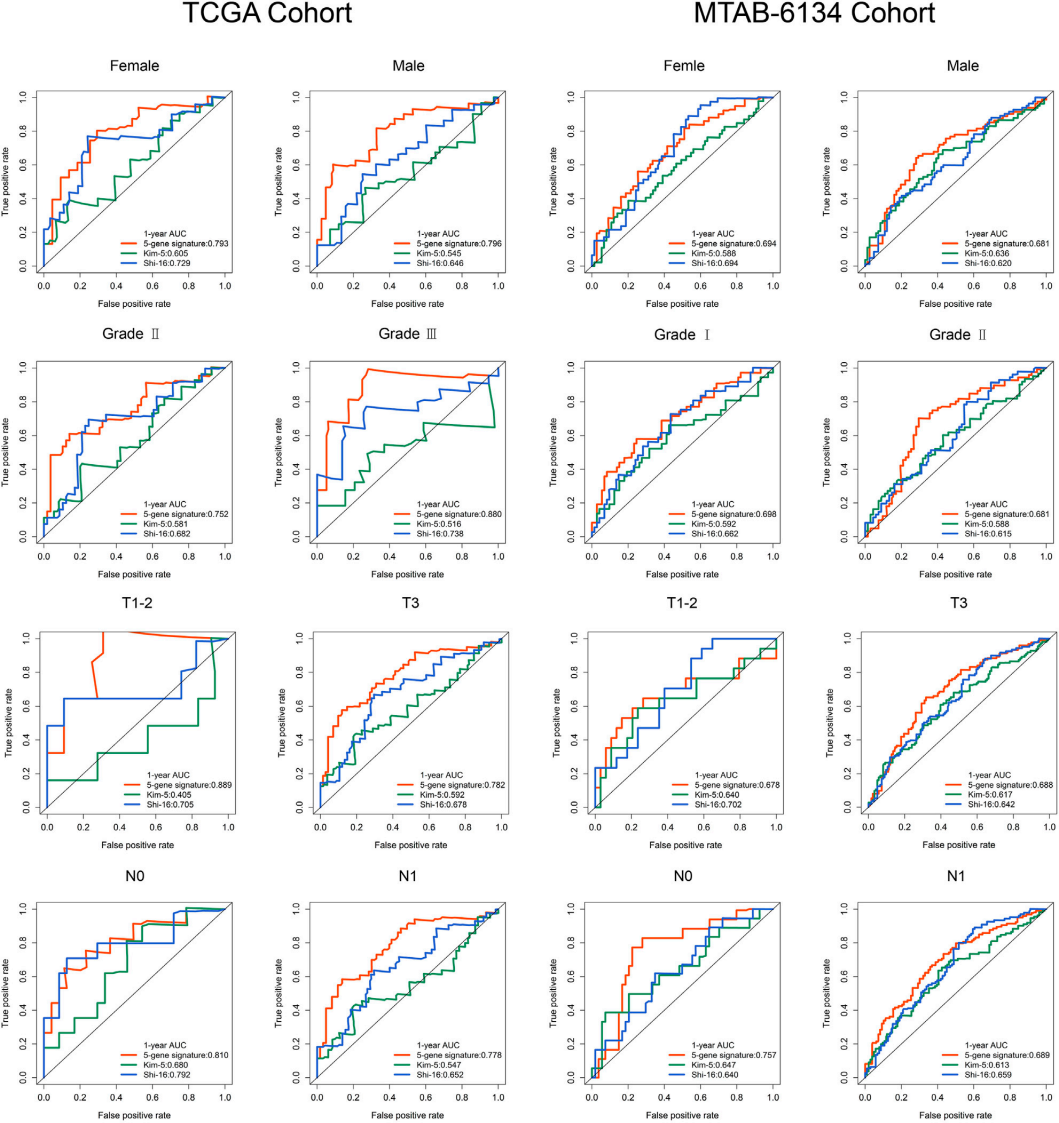
Subgroup analyses evaluate the prognostic stability in TCGA and MTAB-6134 cohorts.

As we know, several clinical variables are important risk factors in PDAC recurrence. To verify the independence and stability of the ferroptosis-related signature, we performed the subgroup analyses through stratifying patients based on gender, histological grade, T stage and N stage. More than half of the patients in PACA-CA cohort have incomplete clinical information and thus TCGA and MTAB-6134 cohorts were selected for further analyses. As shown in Figure 4, no matter what the group was, our signature still had the capacity to predict prognosis with satisfactory accuracy, as the AUC values were no less than 0.75 in TCGA cohort and 0.65 in MTAB-6134 cohort. More importantly, the AUC value of our model was close to or not lower than that of the previous models in each subgroup.

### Predictive Comparison of the Ferroptosis-Related Signature With Clinical Indicators

**FIGURE 5:**
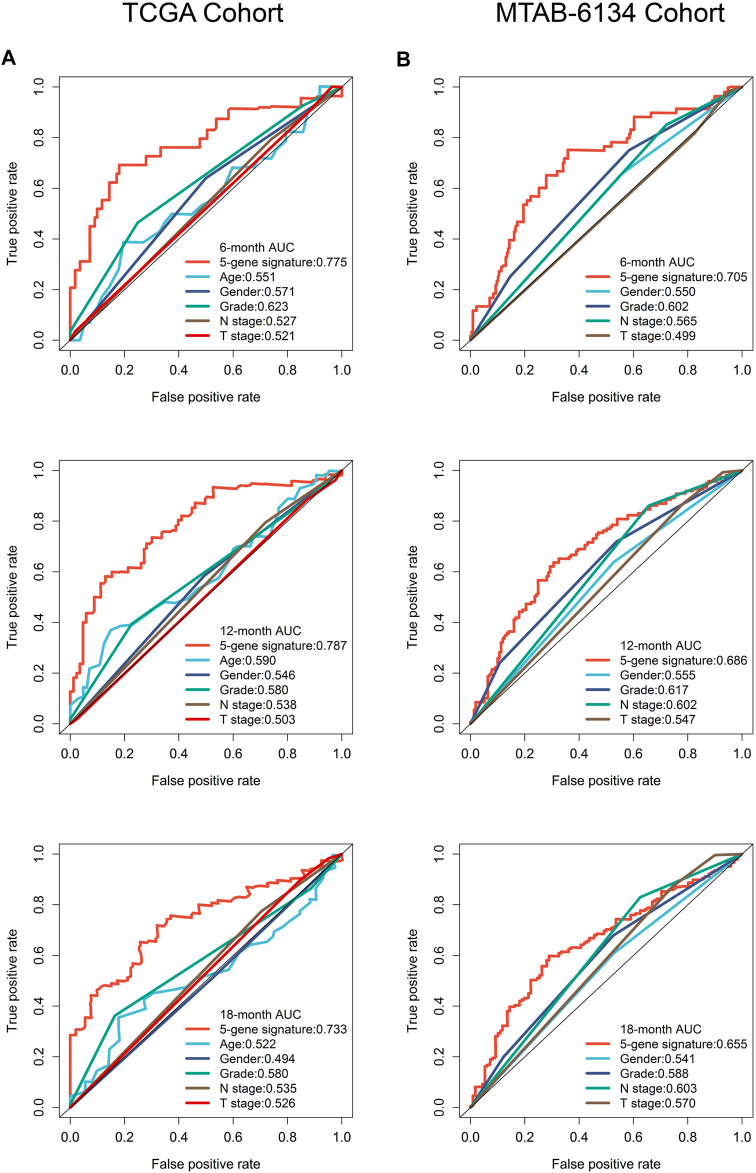
A comparison of the ferroptosis-related signature with clinical indicators. **(A)** ROC curves compared the predictive abilities of the prognostic signature and clinical parameters for 6-months, 12-months and 18-months RFS in the TCGA cohort. **(B)** ROC curves compared the predictive capabilities of the prognostic signature and clinical factors for 6-months, 12-months and 18-months RFS in the MTAB-6134 cohort.

To improve the clinical relevance of this model, we compared the predictive accuracy of the ferroptosis-related signature with that of clinical parameters like grade, T stage and N stage. In TCGA cohort, this signature exhibited more powerful performance in predictive 6-months, 12-months and 18-months recurrence risk compared with traditional parameters (Figure 5A). Similar trends were observed in MTAB-6134 cohort (Figure 5B).

### Enriched Functions and Pathways Related to the Ferroptosis-Related Signature

**FIGURE 6:**
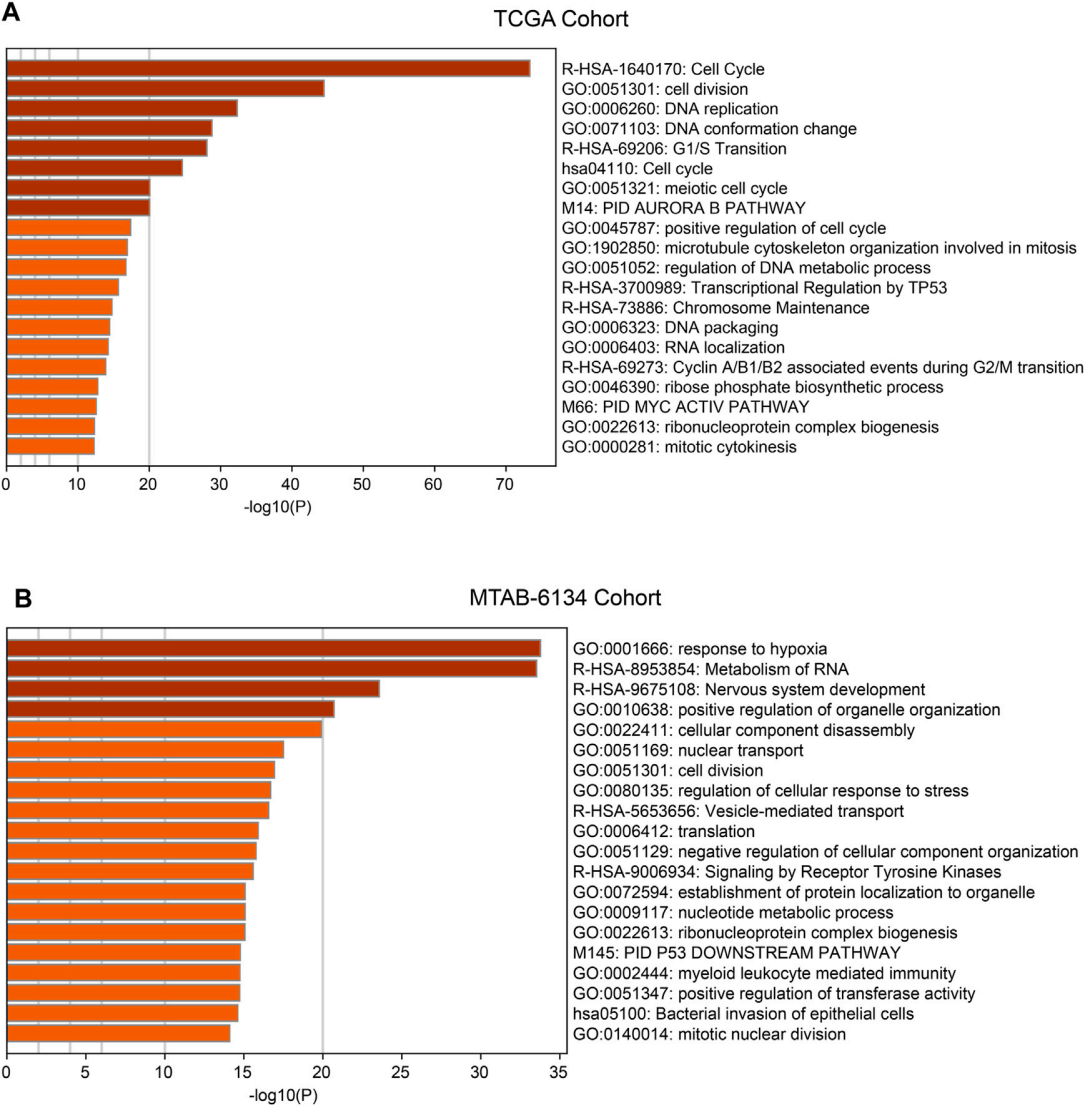
Functions and pathways of the ferroptosis-related signature. **(A)** Functional annotation and pathway enrichment analyses of top 1,000 positively corelated genes of risk model in TCGA cohort. **(B)** Functional annotation and pathway enrichment analyses of top 1,000 positively corelated genes of risk model in MTAB-6134 cohort.

With the aim to preliminarily elucidate biological functions and pathways of the ferroptosis-related signature, we identified top 1,000 genes whose expression were correlated with risk score in TCGA cohort and MTAB-6134 cohort, respectively. The results showed that these genes were involved in several recurrence-related pathways like cell cycle, cell division, DNA replication and TP53-related pathways in two cohorts (Figures 6A,B).

## Discussion

PDAC has an extremely poor outcome even after surgery. Majority of patients develop recurrence after surgical resection (Kim et al., 2019b) and high incidence of recurrence remains the main reason for the unfavorable prognosis of PDAC (Li et al., 2019). In this study, recurrence was observed in 65.7, 80.8 and 61.9% of patients in the TCGA, MTAB-6134 and PACA-CA cohorts, respectively. Accurate recurrence potential prediction of PDAC is of great significance for clinical practitioners to perform specific follow-up strategies and timely intervention of possible recurrence (Daamen et al., 2019). So far, several clinical features have been proved as predictors of recurrence in PDAC, such as resection margin (Ghaneh et al., 2019), tumor size (Ansari et al., 2017), tumor grade (Bilici, 2014) and serum carbohydrate antigen 19‐9 (CA19‐9) (Sugiura et al., 2012). However, few biomarkers and models considering genetic and genomic features of PDAC patients have been identified as postoperative predictors of recurrence in PDAC (Qian et al., 2018).

Ferroptosis, usually regarded as a specific form of regulated cell death, is dependent on the presence of intracellular iron and the accumulation of reactive oxygen species (ROS) (Dixon et al., 2012b). Ferroptosis is mediated by the Fenton reaction, in which Fe2+ reacts with hydrogen peroxide to generate ROS (Xie et al., 2016). ROS are not only a very important secondary signal in cells, but also can also impair the stability of DNA and promote cell death (Dixon and Stockwell, 2014). Variety of molecules have been shown to induce iron degradation and deposition in pancreatic cancer cells, suggesting some new options for the treatment of pancreatic cancer. For example, some researchers have revealed that nuclear receptor coactivator 4(NCOA4)-mediated ferritinophagy, as autophagic process, contributes to ferroptosis via the degradation of ferritin in pancreatic cancer. Degradation of ferritin results in increased intracellular free iron, which triggers ROS generation and consequent ferroptosis in pancreatic cancer (Mancias et al., 2014; Hou et al., 2016). Zhu et al. discovered that heat shock 70 kDa protein 5 (HSPA5) is a negative regulator of ferroptosis in pancreatic cancer cells. HSPA5 is regulated by activating transcription factor 4 (ATF4), and with the activation of ATF4, HSPA5 protein binding to GPX4 increases the stability of GPX4 to protect against ferroptosis. HSPA5-GPX4 pathway make great contribution to regulating ferroptosis in pancreatic cancer (Zhu et al., 2017). In addition to iron metabolism, ROS metabolism also plays a pivotal role in ferroptosis. For instance, ALOX15 can catalyze lipid hydroperoxide generation and ferroptosis in pancreatic cancer (Shintoku et al., 2017), while mitochondrial-targeted nitroxide holds the ability to prevent mitochondrial lipid oxidation on the contrary (Krainz et al., 2016).

In this study, we developed a ferroptosis-related gene signature to predictive recurrence risk in patients with PDAC. This signature exhibited moderate predictive ability with cross-cohort compatibility. In the process of building the signature, we initially performed univariate Cox regression analysis on 260 ferroptosis-related genes and screened 21 survival-related genes. Following multivariate Cox regression analysis identified a 5-gene signature. K-M survival curves confirmed that the signature could effectively capture the RFS differences between low-risk and high-risk groups. ROC analyses demonstrated that the signature had elevated AUC values compared with previously reported models and traditional predictors, indicating a imptoved survival prediction of our model.

Advances in gene chip and high-throughput sequencing have enabled novel development of reliable prognostic models for PDAC patients. To our surprise, there are many models that predict overall survival (Feng et al., 2020; Tan et al., 2020; Wu et al., 2020a; Xu et al., 2021), but few that predict RFS. Meanwhile, few studies have analyzed ferroptosis-related genes expression profiles to construct a risk prediction model. Ferroptosis, a novel form of cell death different from apoptosis, has the potential to promote the efficiency of apoptosis-inducing chemotherapeutic drugs in cell death induction. Thus, ferroptosis inducers are emerging as a promising solution to the problem of tumor drug resistance and combination of ferroptosis inducer and chemotherapy can achieve synergistic reaction and improve the sensitivity of chemotherapy (Stockwell et al., 2017; Shen et al., 2018; Skoupilová et al., 2018). Gemcitabine has become standard of care for postoperative PDAC worldwide and its sensitivity is a key factor affecting tumor recurrence (Liao et al., 2020). Previous studies have proved that the activation of ferroptosis could enhance gemcitabine sensitivity while diminished ferroptosis sensitivity is associated with robust gemcitabine resistance in PDAC (Tang et al., 2020; Ye et al., 2020), indicating that expression of ferroptosis-related gene is a practical tool to predict recurrence.

As for the specific function of these five genes, previous studies have demonstrated that CAV1, DDIT4, SRXN1 and TFAP2C were closely related to PDAC tumorigenesis and progression. CAV1 is overexpressed in PDAC tissues compared with normal tissues and its elevated expression indicates unfavorable prognosis. CAV-1 expression is positively correlated with disease stage, tumor grade and metastatic potential (Huang et al., 2012; Liu et al., 2014). Knockdown of CAV1 results in decreased cell proliferation, invasion, migration and chemotherapy resistance (Chatterjee et al., 2015). DDIT4 is highly expressed in PDAC cells, and DDIT4 inhibition leads to suppressed proliferation, migration, and invasion of PDAC cells (Ma et al., 2020). Expression level of SRXN1 in gemcitabine-resistant SW1990 cells was down-regulated to 6.84-fold change compared with its parental PDAC cell line SW 1990, but the biological role of SRXN1 in PDAC remains unclear (Zhou et al., 2015). Low TFAP2C protein expression is an independent adverse prognostic factor in PDAC (Xiong et al., 2018), while our study proved that high TFAP2C mRNA expression was an unfavorable prognostic indicator. SLC40A1 is a novel iron metabolism associated gene and it regulates iron metabolism to overcome cisplatin resistance in ovarian cancer (Wu et al., 2017; Wu et al., 2020b).

To be honest, there are still many limitations in current research. First, this signature was based on the retrospective data, and need to be verified in more prospective cohorts. Second, all cohorts used in current study have relatively small size (not exceeding 300 patients), partly due to the low opportunities for surgical resection. Our signature needs to be validated in more large-size cohorts in the future. Third, because of the limited number of samples in TCGA and MTAB-6134 cohorts, some subgroup analyses cannot be implemented. In addition, further experiments are needed to clarify the biological implications and relevant mechanisms of selected genes in PDAC progression.

In conclusion, this study is the first to examine ferroptosis-related gene expression profiles in PDAC patients and assess their relationship with tumor recurrence. The proposed signature showed satisfactory performance in predicting PDAC recurrence. The underlying mechanisms of the signature were also been investigated. These results are helpful for improving precision for treatment applications and promoting patient survival and life quality.

## Data Availability

The datasets presented in this study can be found in online repositories. The names of the repository/repositories and accession number(s) can be found in the article/Supplementary Material.

## Abbreviations

AUC, area under the curve; GEO, gene expression omnibus; ICGC, international cancer genome consortium; K-M, Kaplan-Meier; PDAC, pancreatic ductal adenocarcinoma; RFS, recurrence-free survival; ROC, receiver operating characteristic; TCGA, the cancer genome atlas.
